# Transient osteoporosis of the hip

**DOI:** 10.4103/0256-4947.51801

**Published:** 2009

**Authors:** Patricia McWalter, Ahmed Hassan

**Affiliations:** From the Department of Family Medicine, King Faisal Specialist Hospital and Research Centre, Riyadh, Saudi Arabia

## Abstract

Transient osteoporosis of the hip is an uncommon cause of hip pain, mostly affecting healthy middle-aged men and also women in the third trimester of pregnancy. We present a case of transient osteoporosis of the hip in a 33-year-old non-pregnant female patient. This case highlights the importance of considering a diagnosis of transient osteoporosis of the hip in patients who present with hip pain.

Transient osteoporosis of the hip (TOH) was first described in 1959.^1^ The etiology is unknown. More than 200 cases have been reported in the literature, but the disease appears to be rare in the Asian population.^2^ It is known by a variety of terms including bone marrow edema syndrome (BMED), regional migratory osteoporosis, migratory osteolysis, transient hip demineralization and transient osteoporosis. The pathophysiology is unclear. There are at least ten possible causes proposed, which include neurologic, endocrine, vascular and metabolic mechanisms.^3^ In a study from Jeddah, Saudi Arabia, transient osteoporosis of the hip was diagnosed in 7 of 34 consecutive patients presenting with hip pain.^4^ That study showed the importance of magnetic resonance imaging (MRI) when clinical examination is suspect for hip disorder and plain radiographs are normal or equivocal. To our knowledge, it is the only other study from Saudi Arabia discussing TOH/BMED. TOH is self limiting and resolves symptomatically and radiologically within some months of presentation. In women TOH invariably affects the left hip.^1^ Our patient presented with right hip TOH. Our case demonstrates the importance of ruling out other pathologies. TOH is a diagnosis of exclusion. Our patient was managed conservatively and made a good recovery.

## CASE

We report the case of a 33-year-old Saudi female patient who was diagnosed with transient osteoporosis of the right hip. This patient presented to the Family Medicine Department at King Faisal Specialist Hospital and Research Centre, Riyadh in July 2007, with right hip pain radiating down the right leg and difficulty getting up from the sitting and lying positions. She reported straining herself after lifting heavy suitcases. The pain was relieved partially by diclofenac. She had no systemic symptoms like fever or weight loss. She remembered having pain similar to this during her pregnancy 9 years ago. She also described having left gluteal pain 2 years ago, which resolved quickly with simple analgesia. Her right leg pain persisted despite regular non-steroidal anti-inflammatory therapy. The pain was mainly experienced in the right gluteal region and radiated down the right leg to just below the knee. She reported waking at night with the pain. Her medical history included gastric banding surgery three years previously. On examination the patient was apyrexial. There was mild tenderness at L5/S1 and the right sacroiliac joint. She limped, favoring the left side. There was a slight reduction in the range of movement at the lumbar sacral spine. There was some discomfort on internal rotation of right hip but no restriction of movement. Straight leg raising was 80 degrees bilaterally with no neurological findings in the lower limbs. There was some tenderness over the right gluteal region. She found it very painful to weight bear on the right side.

**Figure 1:**
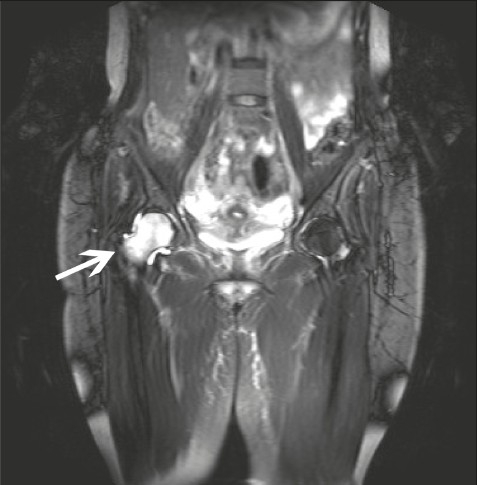
Initial MRI with the classical bone marrow edema picture of the right femoral head and neck.

Laboratory test results included the following: The erythrocyte sedimentation rate (ESR) was 21 mm/h (normal range, 0-10 mm/h). The white cell count and differential were normal. Vitamin D was < 13 nmol/L (normal range, 22-116 nmol/L). Vitamin B12 was 109 pmol/L (normal range, 145-367 pmol/L). Calcium, phosphate, magnesium and alkaline phosphatase were all normal. MRI of the lumbar spine showed facet degenerative changes at L5/S1, but no focal disc herniation. As her symptoms were increasing in intensity MRI of her pelvis was arranged. This showed the following abnormalities: low signal intensity of the right femoral head and neck in T1 images, which was of high signal intensity in T2 and STIR images with some enhancement of contrast medium, particularly in the cranial medial aspect (Figure 1). These findings were suggestive of transient osteoporosis of the right hip. Mild effusion of the right hip was also noted. Incidental findings on the MRI of the pelvis included localized lynphangiomatosis and a small right ovarian cyst. In addition a small area of about 3 cm showing peripheral enhancement in the right gluteal region was noted with the comment an “infectious process is probable”. The patient was referred to the orthopedic clinic urgently to rule out serious pathology. The differential diagnosis at this stage included septic arthritis, malignancy, avascular necrosis (AVN) and transient osteoporosis of the hip.

**Figure 2:**
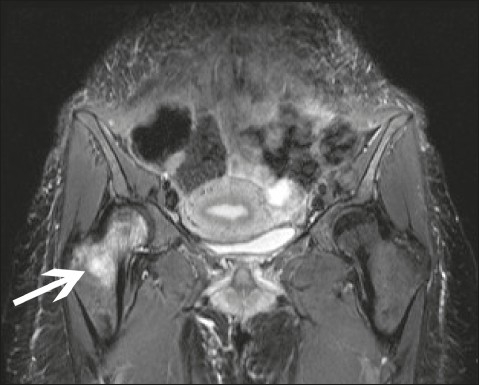
MRI 3 months later showing significant regression of the right hip edema.

The orthopedic consultant did not feel her symptoms were due to septic arthritis as clinical and laboratory investigations did not support this diagnosis. The MRI suggested the presence of a small right gluteal abscess. The patient had received an intramuscular injection of diclofenac on this side when her symptoms first started, which was the likely reason for this appearance as there were no other signs of an abscess clinically. The MRI of the pelvis did not show signs of neoplasia or AVN. The classic appearance of AVN of the hip on magnetic resonance images is that of a local lesion of the femoral head.^6^ The magnetic resonance images of our patient showed diffuse bone marrow edema with an effusion which is rarely seen in avascular necrosis of the hip. The cause of the symptoms was therefore related to TOH. The patient was initially managed with conservative measures including analgesia and strict non-weight bearing. She was prescribed calcium and vitamin D supplements. MRI of the pelvis three months after presentation showed a significant improvement, with regression of the right hip edema (Figure 2). Clinically, her pain had improved but she was advised to restrict herself to partial weight bearing only and to use crutches. One year after her initial presentation her symptoms had completely resolved.

## DISCUSSION

TOH is an uncommon but important cause of hip pain. It was first described in 1959 by Curtis and Kincaid in the third trimester of pregnancy.^6^ It can also affect middle-aged men, and as our case demonstrates, young non-pregnant females. It is also called BMED based on the characteristic MRI findings. Patients present with severe ill-defined unilateral hip pain without a history of trauma. Many are unable to walk without assistance. Pain is generally worse with activity. Physical examination may reveal mild limitation of hip movements with inability to bear weight even if hip pain is minimal.

Laboratory investigations do not help with the diagnosis, but the ESR may be elevated. Radiographic findings may lag behind clinical symptoms by 1 to 2 months.^5^ In contrast, the symptoms of AVN are usually insidious in onset. Functional disability is proportionate to the level of pain. Unlike in TOH, etiological factors can be clearly identified in most patients with AVN of the hip; For example an excessive intake of alcohol and systemic administration of steroids. In our patient, an etiological factor could not be identified. In contrast to transient osteoporosis, AVN is progressive. Transient osteoporosis and AVN of the hip are two separate clinical entities that have several distinguishing clinical and radiographic features.^5^

The hallmark of TOH is its self limiting nature. The natural history of the condition is that of a symptom plateau followed by a gradual resolution of symptoms over 3 to 9 months. Migratory recurrence of symptoms occurs usually within the first 2 years after pain relief.^1^ It is very important to rule out other pathologies like inflammatory arthritis of the hip, AVN, stress fracture of the femoral neck, synovial disorders and neoplasia.^7^ Bone marrow edema can also be seen in hip osteoarthritis and traumatic fractures of the hip. With inflammatory arthropathies of the hip, plain radiographs show that the pattern of joint space loss is predominantly axial in contrast to the superior joint loss typical of osteoarthritis. On MRI, the typical features are of an effusion with synovitis and bone marrow edema on either side.^6^

Patients with AVN usually present with groin or hip pain. Pain may be present for several months and increases in intensity over time. Hip movements are restricted on clinical examination and symptoms tend to correlate with radiological changes. As AVN is bilateral in up to 40% of cases, both hips must be examined simultaneously.^6^ The classic radiographic appearance is that of a mottled radiolucent area surrounded by an area of sclerosis. In the late stages, a radiolucent crescent sign may develop just distal to the articular surface due to subchondral collapse, before flattening of the articular surface.^2^ MRI is the most sensitive and specific imaging technique for detecting transient osteoporosis and osteonecrosis as well as detecting and staging fractures and microfractures.^8^ In patients with septic arthritis, MRI may depict associated marrow edema and suggest its reactive or infectious origin. For the neoplastic disorders, although plain radiographs should be the initial examination, MRI may follow for assessing extension to the surrounding soft tissues and/or associated pathologic fracture, facilitating the treatment planning.^8^ MRI is therefore the investigation of choice to exclude other diagnoses. MRI in TOH shows decreased signal intensity of bone marrow on T1-images and increased signal intensity relative to the intensity of normal marrow on T2-images. Joint effusions are seen on T2-images. An absence of subchondral lesions, delayed peak enhancement of the abnormal marrow on perfusion images and sparing of the subchondral zone from marrow oedema are MRI findings highly correlated to TOH.^9^ The absence of focal changes on MRI is highly suggestive of a transient lesion.^10^ A greater level of awareness of this condition is needed to differentiate TOH from AVN, avoiding unnecessary surgery and ensuring appropriate treatment.^10^ A careful and systematic analysis of the MR images is recommended when facing BMED of the femoral head, given the variable outcomes observed in the numerous conditions showing this syndrome.^11^

We agree with Ragab et al, who also studied BMED in Saudi Arabia, that early diagnosis and treatment of hip pain is important and MRI is the modality of choice.^4^ Our findings concur with Diwanji et al on the conservative treatment of patients with TOH. Patients correctly diagnosed as having TOH usually recover well on conservative treatment.^2^ Antiresorptive agents, including calcitonin and bisphosphonates, have been reported to alleviate pain and accelerate clinical and radiological recovery, although these data come from individual case reports and uncontrolled case series.^12^ In the absence of clinical trials demonstrating the effectiveness of these agents, it seems reasonable to initially treat TOH conservatively, and reserve the use of antiresorptives for those with severe pain or disability, and those at highest risk of fracture.^11^

In conclusion TOH is managed with analgesia, physical therapy and protected weight bearing. This is essential to avoid stress fractures. It is important to consider TOH in the differential diagnosis of middle-aged men and young women with hip pain.
